# Dihydroartemisinin as a Putative STAT3 Inhibitor, Suppresses the Growth of Head and Neck Squamous Cell Carcinoma by Targeting Jak2/STAT3 Signaling

**DOI:** 10.1371/journal.pone.0147157

**Published:** 2016-01-19

**Authors:** Lifeng Jia, Qi Song, Chenyang Zhou, Xiaoming Li, Lihong Pi, Xiuru Ma, Hui Li, Xiuying Lu, Yupeng Shen

**Affiliations:** 1 Postgraduate School, The Third Medical Military University, Chongqing, 400038, China; 2 Department of Otolaryngology Head and Neck Surgery, Bethune International Peace Hospital, Shijiazhuang, 050081, Hebei Province, China; 3 Postgraduate School, Medical College of PLA, Beijing, 100700, China; 4 Department of Otolaryngology, Hebei General Hospital, Shijiazhuang, 050051, Hebei Province, China; 5 Department of Basic Sciences, Hebei College of Traditional Chinese Medicine, Shijiazhuang, 050061, Hebei Province, China; 6 Department of Pathology, Bethune International Peace Hospital, Shijiazhuang, 050081, Hebei, Province, China; Yong Loo Lin School of Medicine, National University of Singapore, SINGAPORE

## Abstract

Developing drugs that can effectively block STAT3 activation may serve as one of the most promising strategy for cancer treatment. Currently, there is no putative STAT3 inhibitor that can be safely and effectively used in clinic. In the present study, we investigated the potential of dihydroartemisinin (DHA) as a putative STAT3 inhibitor and its antitumor activities in head and neck squamous cell carcinoma (HNSCC). The inhibitory effects of DHA on STAT3 activation along with its underlying mechanisms were studied in HNSCC cells. The antitumor effects of DHA against HNSCC cells were explored both in vitro and in vivo. An investigation on cooperative effects of DHA with cisplatin in killing HNSCC cells was also implemented. DHA exhibited remarkable and specific inhibitory effects on STAT3 activation via selectively blocking Jak2/STAT3 signaling. Besides, DHA significantly inhibited HNSCC growth both in vitro and in vivo possibly through induction of apoptosis and attenuation of cell migration. DHA also synergized with cisplatin in tumor inhibition in HNSCC cells. Our findings demonstrate that DHA is a putative STAT3 inhibitor that may represent a new and effective drug for cancer treatment and therapeutic sensitization in HNSCC patients.

## Introduction

Signal transducer and activator of transcription (STAT) proteins are a family comprised of seven members, includingSTAT1, STAT2, STAT3, STAT4, STAT5a, STAT5b, and STAT6. It is now clear that STAT3, originally considered as an acutephase response protein [1], is a latent cytoplasmic protein that can be activated by various extracellular polypeptides and other stimuli. These include cytokines (such as IL-6) and growth factors (such as EGF) [2], and hypoxia stress [3], etc. Activation of STAT3 involves phosphrylation of specific tyrosine on STAT3, which in turn induces STAT3monomer’s homodimerization and/or heterodimerization with STAT1 or STAT5 through reciprocal Src homology 2 (SH2) domain/phosphotyrosine interactions. Consequently, dimerized STAT3 translocates to the nucleus and binds to specific DNA sequences, and regulates transcription and expression of downstream genes that are associated with cell survival and proliferation [4], cell cycle regulation [5], apoptosis [6] and angiogenesis [7].

Persistent phosphorylation of STAT3 has been found in numerous malignant neoplasms [8–14], such as head and neck cancers [8]. In fact, frequency of persistentSTAT3activation is more than 95% in head and neck cancers. It has been reported that activation of STAT3 plays a crucial and pivotal role in initiation of malignant transformation [9], immune evasion and suppression [10], as well as cancer invasion and metastasis, suggesting that therapeutic interventions specifically targeting STAT3 can convert the effects of STAT3 activation from pro-tumor to anti-tumor events. Therefore, several novel small-molecule compounds have been developed to inhibit STAT3 phosphorylation [11, 12], but their poor solubility and ambiguous after-effects to host preclude them from clinical trials and practical uses in cancer treatment.

Dihydroartemisinin (DHA) is a semi-synthetic derivative and main active metabolite of the artemisinin, a natural product isolated from a Chinese medicinal herb (Artemisia annua). It is one of first-line antimalarial drugs recommend by World Health Organization in regions where Plasmodium falciparumis becomes resistant to traditional drugs. Moreover, DHA has been shown to exert antibacterial [13] and antiviral [14] effects. In addition to these efficacies, evidence from epidemiological, pharmacological and case control studies has suggested that DHA possess antitumor activity and selective cytotoxicity to various malignancies [15–18]. Notably, its low toxicity to host and easy solubility in water is the major incentive for developing the compound as an anticancer agent.

A most recent preliminary study [19] revealed that in T cells of contact hypersensitivity mouse model, artemisinin exerted a strikingly inhibitory effect on IL-17 production, and diminished the level of IL-6, which effects were accompanied with a significant reduction of STAT3 activation, suggesting that reduced STAT3 activation is a result of IL-16 expression inhibition. However, there is no further and direct evidence for proving artemisinin/DHA to be a putative STAT3 inhibitor, and little is known about inhibition effects of DHA on proliferation of HNSCC cells. In the present study, we tested the possibility of DHA as a putative STAT3 inhibitor. Therapeutic potency of DHA against HNSCC cells was validated in vitro and in vivo. It is for the first time that we identified DHA as a putative inhibitor of STAT3, and thus the compound represents a promising therapeutic agent against HNSCC.

## Materials and Methods

### Cell lines and main reagents

Human HNSCC Fadu and Hep-2 cells were obtained from the American Type Culture Collection (Manassas, VA). Cal-27 cells were purchased from American Type Culture Collection (Manassas, VA). The cells were maintained in Dulbecco’s modified Eagle’s medium or RPMI-1640 medium supplemented with 10% fetal bovine serum (Gibco, Rockville, MD) and 1% penicillin and streptomycin (Gibco, Rockville, MD)under conditions of 37°C, 5% CO_2_, and 95% humidity in a carbon dioxide incubator. DHA was provided by Tokyo Chemical Industry, Co, Ltd (Tci, Tokyo, Japan), which was dissolved in dimethyl sulfoxide (DMSO) (Sigma, St. Louis, MO)and stored as a 200mmol/L stock solution and frozen in aliquots at −20°C. Monoclonal antibodies to p-Jak2(Tyr1007/1008), Jak2, p-SRC (Tyr416), p-EGFR(Tyr1068), p-Akt (Ser473), p-Stat3(Tyr705), Stat3, p-ERK1/2 (Thr202/Tyr204), Bcl-xl, CyclinD1, Mcl-1, MMP-2, and MMP-9 were obtained from Cell Signaling Technologies (Cambridge, MA). Polyclonal antibody to HIF-1α was purchased from Abcam (Cambridge, MA). Polyclonal antibody to VEGF was provided by Santa Cruz Biotechnology (Santa Cruz, CA). Monoclonal antibody to Anti-β-actin was purchased from Bioworld Technology Inc (St. Louis Park, MN, USA). IL-6 was a product from PeproTech (Rocky Hill, NJ, USA).

### Western blot analysis

HNSCC cells from culture or from tissue specimens were lysed in Radio Immunoprecipitation Assay (RIPA) lysis buffer (Beyotime Institute of Biotechnology, Haimen, China) and Western blotting was performed using previously described procedures [20]. Briefly, equivalent amounts of proteins were separated by 10% or 12%SDS-PAGE, and then transferred onto a PVDF membrane (Milipore Corporation, Temecula, CA). After blocking with TBS plus 5% non-fat milk for 1 h at room temperature, the membrane was incubated with indicated primary antibodies overnight. Membranes were incubated with a horseradish peroxidase-conjugated secondary antibody (Zhongshan Goldenbridge Co, Ltd, Beijing, China) for 1 h at room temperature. Immunoreactive proteins were visualized with an enhanced chemiluminescence detection system (GE Healthcare Life Sciences, Amersham, UK).

### MTT assay

In brief, cells were seeded in 96-well culture plates (10×10^3^cells /200 μl per well) and incubated overnight, and then treated for 24 or 48h with various concentrations of DHA or with the diluting vehicle (DMSO). After incubation, freshly prepared 3-(4,5-dimethylthiazol-2-yl)-2,5-diphenyltetrazolium bromide (MTT) (20μl; 5 mg/ml; Sigma) was added to each well and incubated for 4 h at 37°C. The culture medium was removed from the wells and substituted with the reduced MTT solublized in 150μl/well DMSO. The prepared plates were subjected to examination with an Enzyme-linked Immunosorbent Detector (Model 550, Bio-Rad, Hercules, CA, USA) with absorbance values of 490 nm to evaluate cell viability.

### Wound healing assay

1×10^5^HNSCC cells were seeded in 24-well plates and starved for 12 h in serum-free medium. Then a scratch in the cell monolayer was made using a sterilized pipette tip (10μl). Thereafter, all wound-healing processes were performed in serum-free conditions. After washing three times with 0.01M phosphate-buffered saline (PBS, pH 7.4), the plates were incubated with DHA (40 or 20μM) or with the DMSO, and images were captured at 0, 12, 24 and 48 h using digital camera (Canon, Tokyo, Japan) connected to an inverted microscope (Olympus, Tokyo, Japan). The distance of wound sealing was calculated with Image J software (National Institutes of Health, MD, USA).

### Plasmid constructs and transient transfections

Dominant negative EGFR (DN-EGFR, GenBank_ID: NM_005228), dominant negative Jak2 (DN-Jak2, GenBank_ID: NM_004972), and dominant negative SRC (DN-SRC, GenBank_ID: NM_005417) plasmid constructs were purchased from Gene Chem Co, Ltd (Shanghai, China), and detected by Reverse transcription polymerase chain reaction (RT-PCR) and Western blotting (Figs A and B in S1 File). A constitutive active STAT3 (CA-STAT3, GenBank ID: NM_139276) construct was obtained from Gene Copoeia, Inc (Guangzhou, China). 3×10^5^Cal-27 cells per well were seeded in 6-well plates. When plated cells reached 80% confluence, transfections were performed with Lipofectamine 2000 (Invitrogen Corp, Carlsbad, CA) according to the manufacturer’s instructions. After 24 h, the transfected cells were ready for subsequent DHA treatment studies.

### Flow cytometry

4×10^5^ HNSCC cells were seeded in each well of 6-well plates in triplicates, incubated overnight, and treated with various concentrations of DHA, or DMSO for 24 h. For cell cycle analysis, the treated cells were harvested, washed twice with ice-cold PBS and fixed in 70%methanol, the samples were exposed to RNase A and stained with propidium iodide (PI) for analyzing the DNA content by FACS Calibur flow cytometer (Becton Dickinson, San Diego, CA). For assessing cell apoptosis, we used the Annexin V-FITC Apoptosis Detection Kit (Becton Dickinson, San Diego, CA) and performed the assessment according to manufacturer’s instructions. Percentage of apoptotic cells was analyzed by FACS Calibur flow cytometer.

### Murine xenograft model and tumor treatment

Four to 6weeks old BALB/c male mice weighing18 to 20 g were obtained from Vital River Laboratory Animal Technology Co. Ltd. (Beijing, China) and were maintained in an air-conditioned room with constant temperature (22–24°C) and a dark-light cycle (12 h/12 h), and housed in plastic cages, maximum 5 mice per cage. They were fed a standard chow and tap water ad libitum. All animal experiments were reviewed and approved by the ethics committee of Bethune International Peace Hospital. To establish a xenograft tumor, 1×10^7^ Cal-27 cells in 200 μl culture medium were inoculated subcutaneously into the left inguinal area of each nude mouse. The general conditions of animal, including mental state, diet and defecation were observed every day after tumor implantation. Furthermore, the bitten wound and locally cutaneous ulcer were also paid close attention. Tumor-bearing mice were randomly assigned to either treatment group or control group when average tumor diameter reached 5 mm. The treatment group received intraperitoneal injection of DHA at a dosage of 50 mg/kg once daily, 5 times per week, for 4 weeks. The control group was given DMSO. Tumor size and body weight was measured every 4days throughout the study. Tumor volume was calculated by the formula: V (cm^3^) = width^2^ (cm^2^) × length cm)/2. Tumor growth inhibition rates were calculated using the formula (1-average tumor weight of experimental group/average tumor weight of control group) × 100%. In our experiments, no mice were observed to be died of tumor loading. All animals were pre-euthanized with CO_2_ and sacrificed by cervical dislocation at the termination of experiments, and the tumors were excised and weighed. The portions of each tumor were taken for Western blot and pathological analyses.

### Statistical analysis

All in vitro experiments were repeated at least three times and data were expressed as mean ± SD. Student’s t test or on-way ANOVA was used for statistical analysis when indicated. *P* values < 0.05 were considered to indicate statistical significance.

## Results

### DHA, as a putative STAT3 inhibitor, effectively blocks activation of STAT3 in HNSCC cells

**Fig 1:**
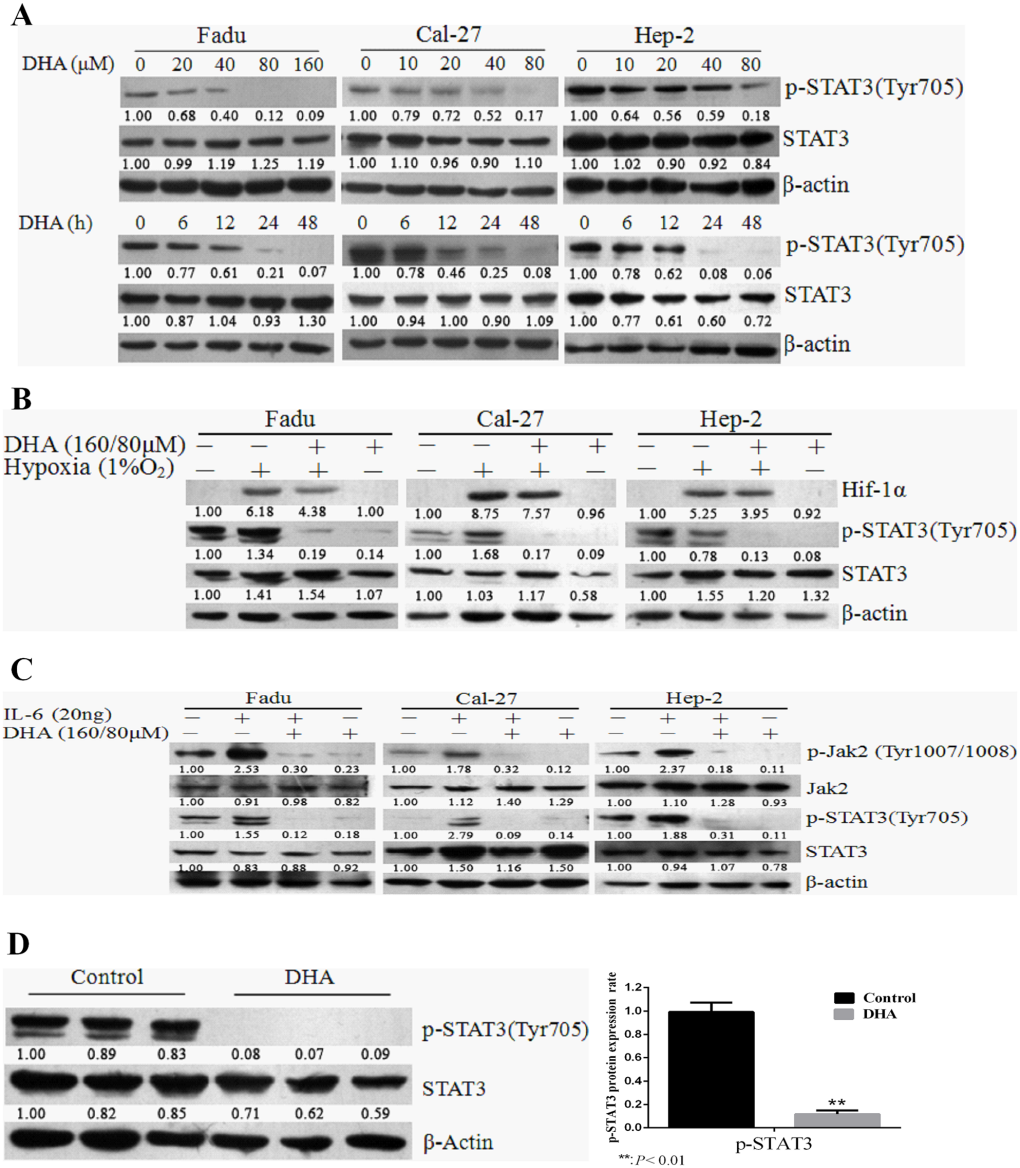
DHA selectively blocked activation of STAT3 under different conditions in HNSCC cells. (A) DHA blocked constitutive activation of STAT3. FaDu, Cal-27, and Hep-2 cells incubated with indicated concentrations of DHA or DMSO for 24 h (upper panel), or treated with fixed concentrations (160 μM for Fadu cells, and 80μM for Cal-27 and Hep-2 cells) of DHA for 0, 12, 24, and 48 h (lower panel). Expression of p-STAT3 was determined by Western blotting. (B) DHA inhibited hypoxia-induced activation of STAT3. Three HNSCC cell lines were treated with DHA (160 μM for Fadu, and 80μM for Cal-27 and Hep-2) under hypoxia for 24 h. Levels of p-STAT3and HIF-1α were determined by Western blotting. (C) DHA blocked IL-6-induced activation of STAT3. HNSCC cells were treated with160 μM DHA (Fadu) or 80 μM DHA (Cal-27 and Hep-2) for 24h and exposed to IL-6 (20ng) for 1 h. Levels ofp-Jak2 and p-STAT3 were evaluated by Western blotting. (D) DHA inhibited STAT3 activation in vivo. Tumor-bearing mice were treated with DHA as described in the materials and methods. Expression of p-STAT3 in representative tumor tissues of experimental and control animals was evaluated by Western blotting. All experiments were performed in triplicates.

First, DHA inhibits constitutive phosphorylation and activation ofSTAT3 in HNSCC cells. As observed, three HNSCC cell lines (Fadu, Cal-27 and Hep-2) expressed certain amount of p-STAT3 under normal cultural conditions, typical of constitutive or persistent activation of STAT3 (S1 Fig). DHA significantly inhibited activation of STAT3 in the three HNSCC cell lines in dose- and time-dependent manners (Fig 1A). Second, DHA effectively inhibited activation of STAT3 induced by hypoxia and IL-6 in HNSCC cells. When hypoxia or IL-6 was given, activation of STAT3 was substantially induced, which was effectively inhibited by DHA (Fig 1B and 1C). Finally, inhibition of STAT3 activation by DHA was also confirmed in vivo as observed on expression of p-STAT3 in the xenograft animal tumors (Fig 1D). Taken together, DHA inhibits STAT3 activation under different circumstances.

**Fig 2:**
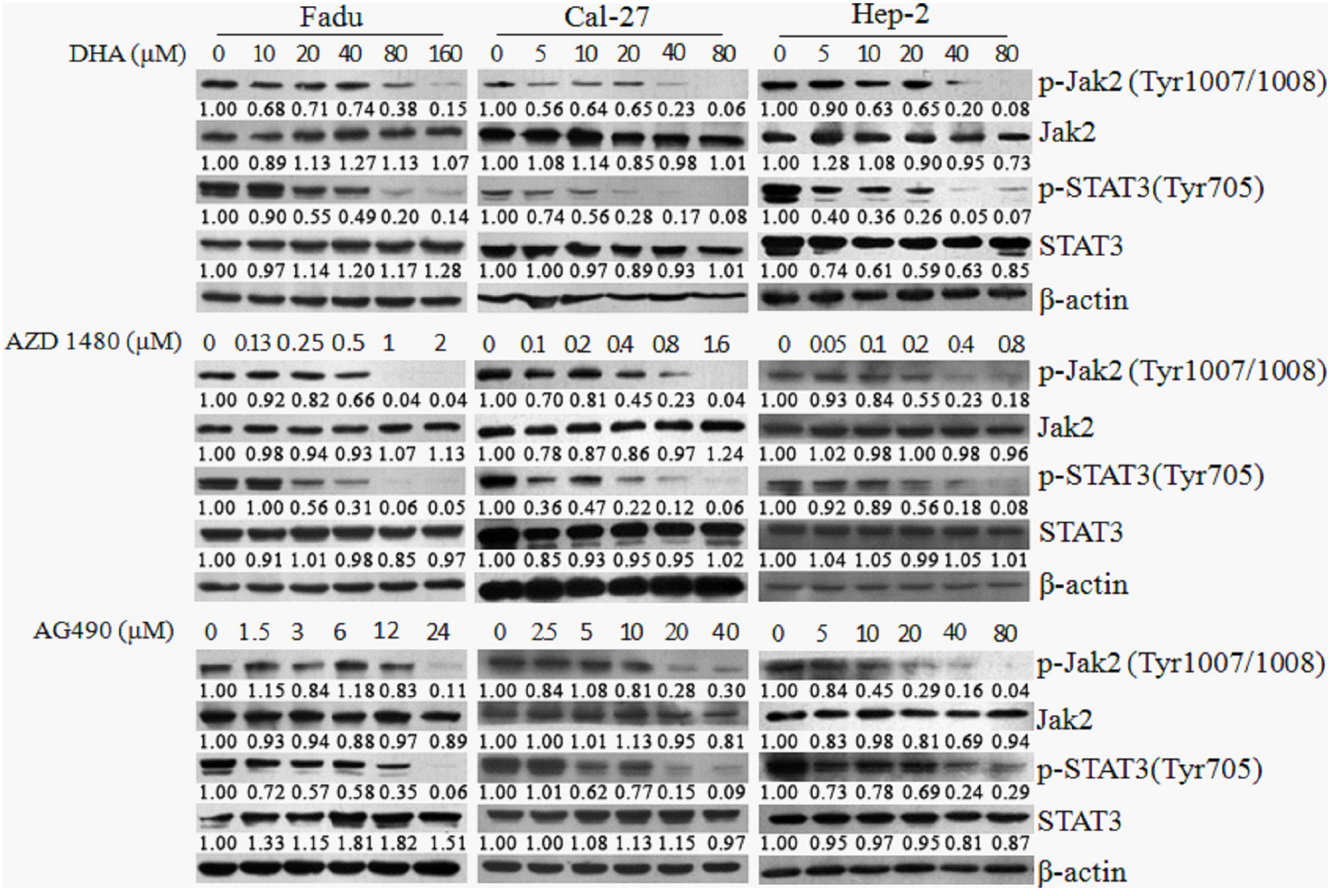
Comparison of inhibitory effect of DHA on STAT3 activation with that of other two available Jak/STAT3 inhibitors. Fadu, Cal-27 and Hep-2 cells were exposed to different concentrations ofDHA, AZD1480 and AG490 or DMSO for 24 h, after which time the expression of p-Jak2 and p-STAT3 were analyzed by Western blotting. The doses of these inhibitors were chosen based on a preliminary dose-escalation study (S3 Fig).

The efficacies of DHA on STAT3 inhibition were compared with those of AZD1480 and AG490, two specific inhibitors of Jak2/STAT3 signaling, which are commonly used in the experimental studies. The doses of AZD 480 and AG490 were chosen based on a preliminary dose-escalation study (S2 Fig). All of the three inhibitors remarkably downregulated levels of p-Jak2 and p-STAT3in HNSCC cells (Fig 2). The inhibitory effects of DHA on STAT3 activation were comparable with those of AZD 480 and AG490, which was most likely more effective than that of AG490 in Hep-2 cells (Fig 2, the right panel).

### Inhibition of STAT3 activation by DHA in HNSCC cells is attributed to selective blockade of Jak2 phosphorylation

**Fig 3:**
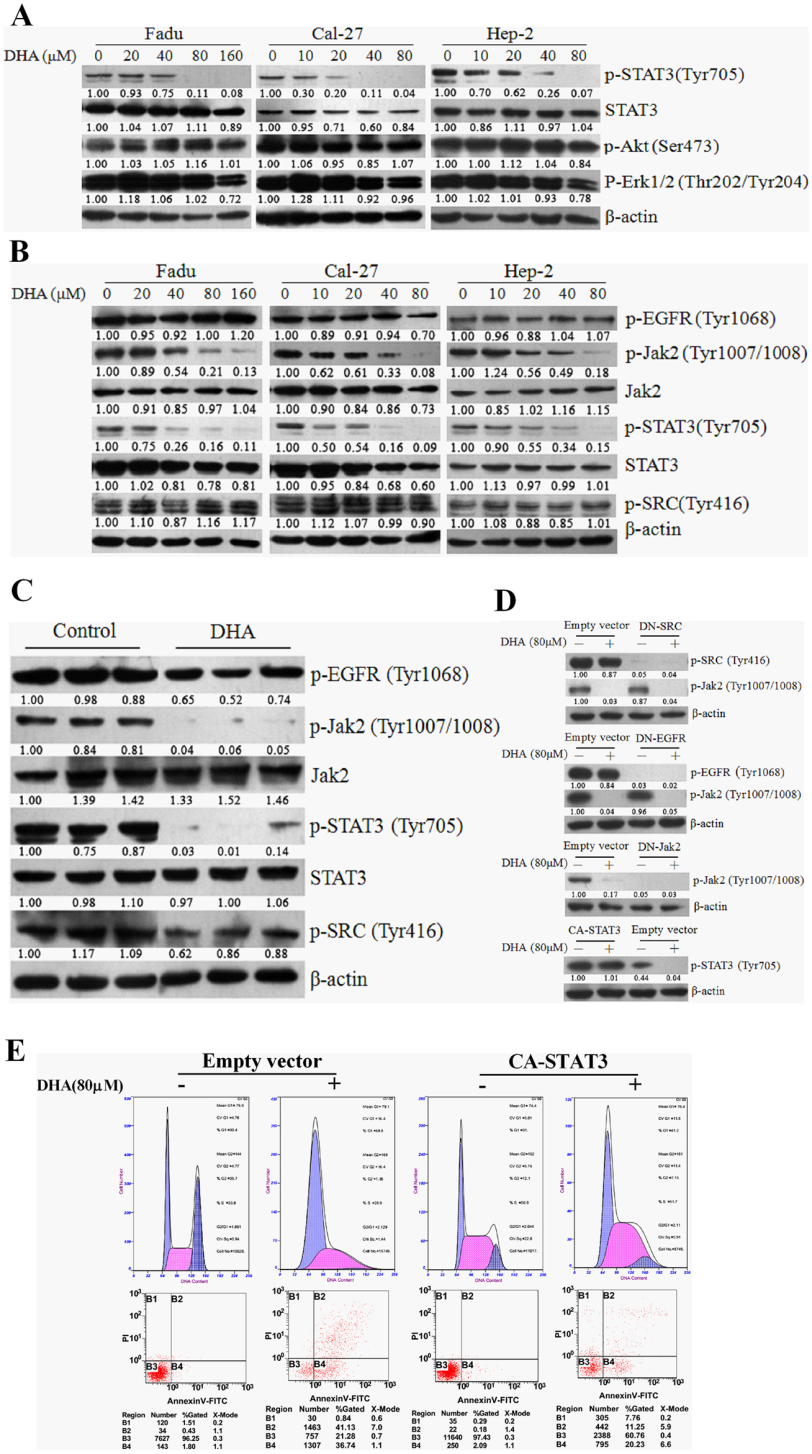
Inhibiton of STAT3 activation by DHA in HNSCC cells was mediated by selective blockade of Jak2 phosphorylation. (A) DHA inhibited STAT3 activation, but did not affect the expression of p-Erk1/2 and p-Akt. FaDu, Cal-27 and Hep-2 cells were treated with indicated concentrations of DHA or DMSO for 24 h. Western blotting was used to determine the levels of p-STAT3, p-Erk1/2 and p-Akt. (B) DHA inhibited expression of p-Jak2 and p-STAT3, but not of p-EGFR and p-SRC. FaDu, Cal-27 and Hep-2 cells were treated with indicated concentrations of DHA for 24h. Expression of the associated proteins was detected by western blotting. (C) DHA selectively blocked Jak2/STAT3 activation in vivo. Tumor-bearing mice were treated with DHA as described. Expression of p-EGFR, p-Jak2, p-SRC and p-STAT3 in tumor tissues was analyzed by Western blotting. (D) DHA specifically blocked Jak2 activation in HNSCC cells. Cal-27 cells were transfected with DN-EGFR, DN-Jak2, DN-SRC, CA-STAT3, or empty vector, and exposed to 80 μM DHA for 24 h. Expression of p-EGFR, p-Jak2, p-SRC and p-STAT3 were studied by Western blotting. (E) CA-STAT3attenuated the cell cycle arrest induced by DHA. Cal-27 cells were transfected withCA-STAT3or empty vector and exposed to 80 μM DHA for 24 h. Cell cycle and cell apoptosis were analyzed by flow cytometry. All experiments were performed in triplicates.

After treating Fadu, Cal-27 and Hep-2 cells with different concentrations of DHA, inhibition of STAT3 activation was prominent, but little or no inhibition of Akt and ERK phosphorylation was observed (Fig 3A); the expression of p-Jak2 in three HNSCC cell lines was markedly reduced in a dose-dependent manner; however, neither inhibition of p-SRC nor downregulation of p-EGFR was observed (Fig 3B). It was of particular interest to note that, in the xenograft animal tumors, application of DHA resulted in significant simultaneous decrease of p-STAT3 and p-Jak2 expression, but the levels of p-SRC and p-EGFR remained unchanged (Fig 3C). Furthermore, treatment with DHA and/or dominant negative Jak2 (DN-Jak2) rather than abrogation of EGFR and SRC activation by their dominant negative constructs (DN-EGFR and DN-SRC) reduced the expression of p-Jak2 and p-STAT3 in Cal-27 cells (Fig 3D). However, DHA did not altered the level of overexpressed p-STAT3, apoptosis, and cell cycle in Cal-27 cells transfected with constitutively active STAT3 (CA-STAT3) construct (Fig 3E). Collectively, inhibition of STAT3 activation by DHA is through selective blockade of Jak2 phophorylation and activation.

### DHA inhibits proliferation, growth and migration of HNSCC cells in vitro

**Fig 4:**
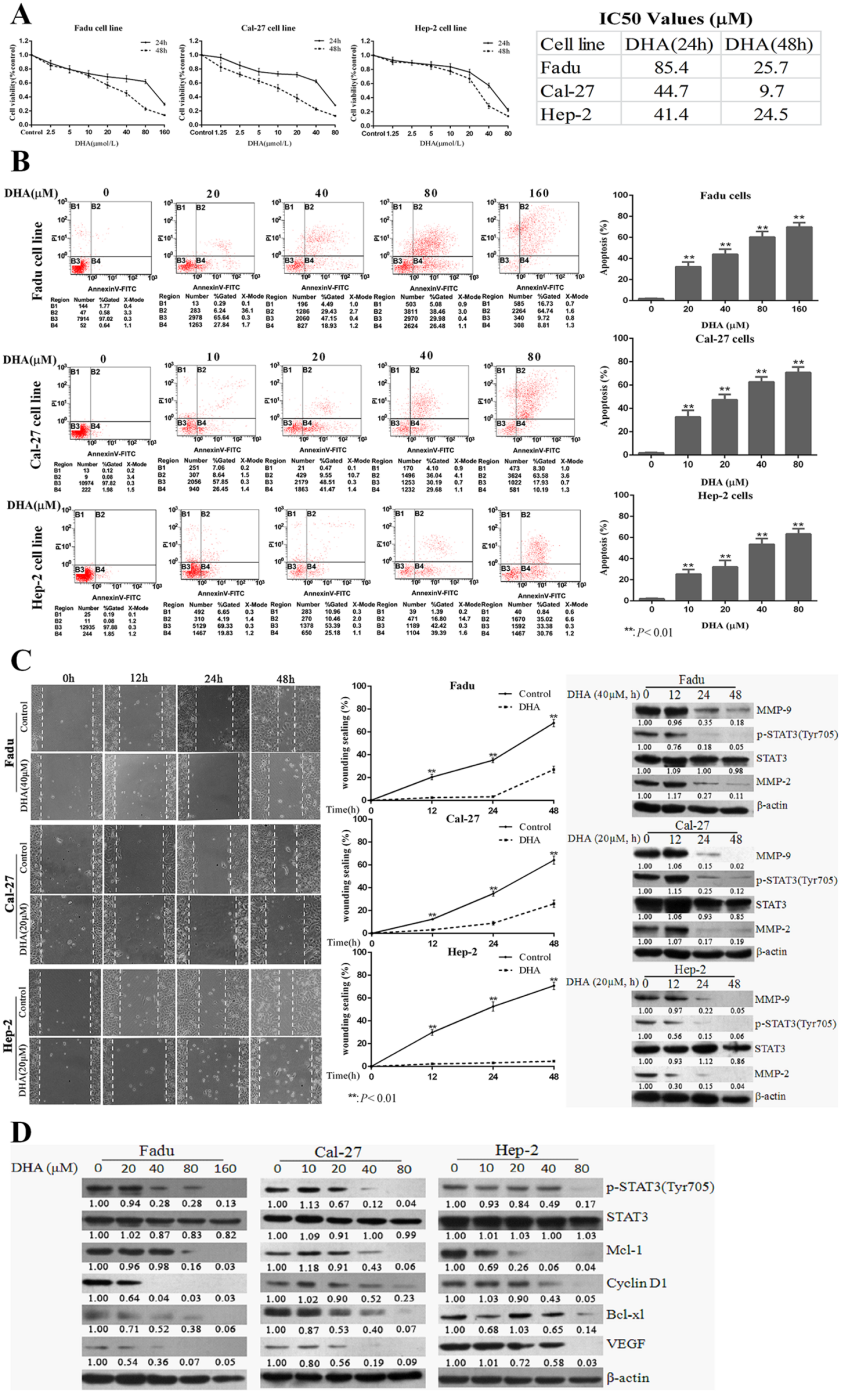
DHA resulted in proliferation inhibition and migration, and induced apoptosis in HNSCC cells. (A) FaDu, Cal-27, and Hep-2 cells were treated with indicated concentrations of DHA for 24 or 48h, and cell viability was tested via MTT assay. IC50 values of DHA were calculated for the three cell lines. (B) DHA induced apoptosis in HNSCC cell. Three HNSCC cell lines were incubated with indicated concentrations of DHA for 24 h, followed by flow cytometric analysis with Annexin V-FITC and propidium iodide (PI) labeling. (C)DHA induced inhibition of migration of HNSCC cells. Cells were incubated with 40μM (FaDu) or 20μM (Cal-27and Hep-2) DHA or DMSO. The wound healing capacity was measured at 0, 12, 24, and 48 h. Data were expressed as means ± SD (left and middle panels). Simultaneous determination of levels of MMP-2, MMP-9 and p-STAT3 was conducted (right panel). (D) Effects of STAT3 inhibition by DHA on expression of downstream proteinsMcl-1, Bcl-xl, Cyclin-D1 and VEGF in HNSCC cells as determined by Western blotting. All experiments were performed in triplicates.

DHA significantly inhibited the proliferation of HNSCC cells. Fadu cells were the least sensitive to DHA while other two cell lines were more sensitive to the compound comparatively (Fig 4A). The DHA-induced growth inhibition involved apoptosis induction. The percentage of the apoptotic cells in HNSCC cells treated with DHA was greater than that in untreated (control) cells (Fig 4B). It was also observed that DHA reduced the migration of all three cell lines in a time-dependent manner (Fig 4C, left and middle panels) (p<0.01). As demonstrated in the cell migration assay, expression of two important metastasis-associated proteins (MMP-2 and -9), were remarkably decreased after treatment with DHA (Fig 4C, right panel). Other proteins downstream of STAT3 including Mcl-1, Bcl-xl, Cyclin-D1 and VEGF were also downregulated in the three HNSCC cell lines after DHA treatment (Fig 4D).

### DHA inhibits tumor growth in the xenograft animal models of human HNSCC

**Fig 5:**
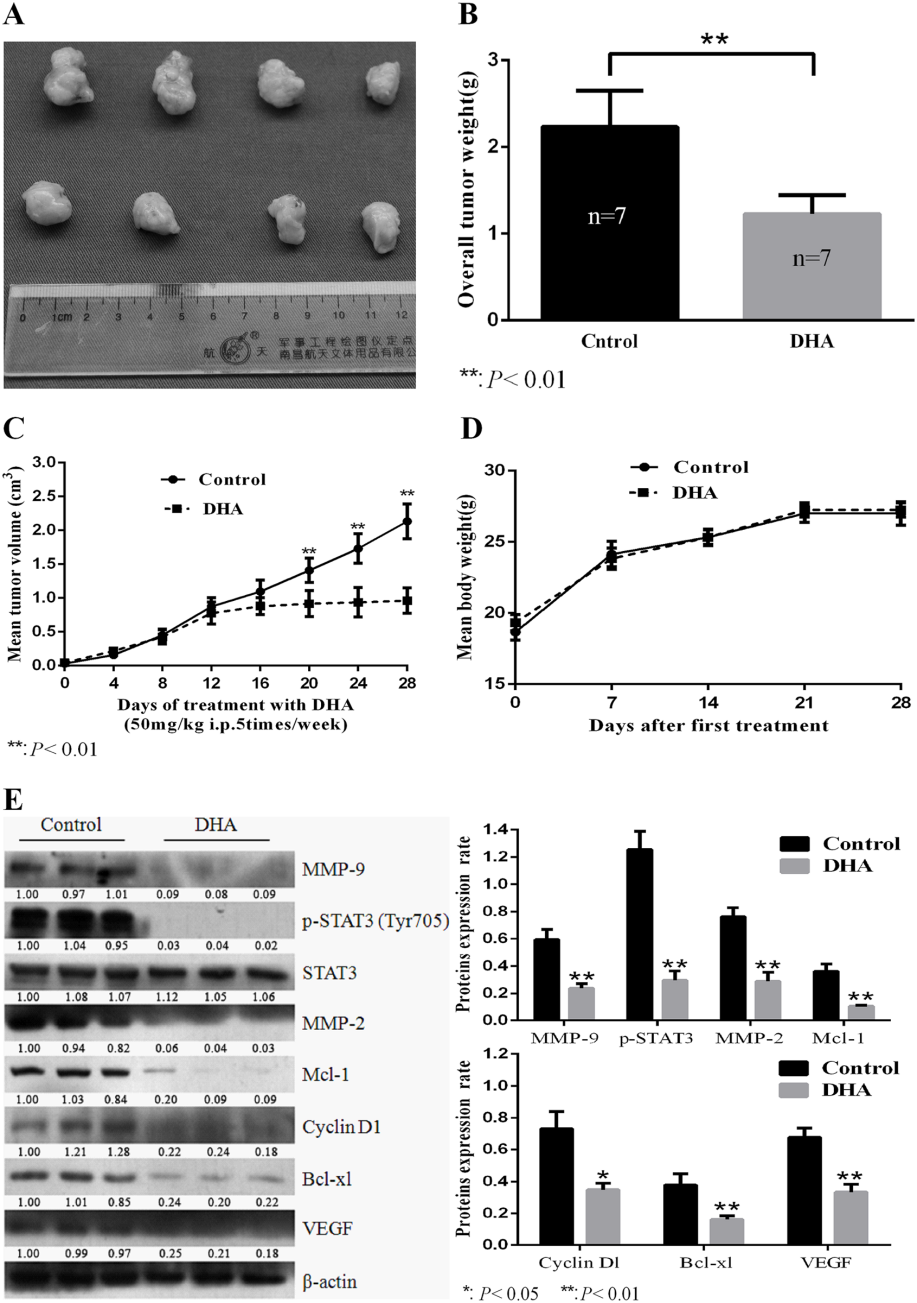
DHA inhibited growth of HNSCC in vivo. (A) Cal-27 cells were used to establish xenograft tumors in BALB/c mice, and animals were treated with DHA or vehicle (DMSO) as described. Representative xenograft tumors from DHA-treated mice and vehicle-treated mice were presented. (B) Overall weight of the dissected tumors. Data were expressed as means ± SD (n = 7), **p<0.01. (C) The changes of mean tumor volume in DHA-treated mice and vehicle-treated mice. Data were expressed as means ± SD (n = 7), **p<0.01. (D) The dynamic body weight changes of tumor-bearing mice during DHA treatment. (E) Effects of DHA on the downstream proteins ofJAK2/STAT3 pathway in xenograft tumors as demonstrated by Western blotting. Data were expressed as means ± SD (n = 7), *p<0.05, **p<0.01. All experiments were performed in triplicates.

Compared to the vehicle (DMSO), DHA significantly decreased tumor size, overall tumor weight, and mean tumor volume (Fig 5A–5C). However, the general conditions and the body weight of the animals treated with DHA showed nearly no change for the period of DHA application, which implies that the compound has no obvious toxicity to experiment animals (Fig 5D). As expected, the expressions of the above-mentioned functional proteins downstream of STAT3 were all inhibited in xenograft animal tumors treated with DHA. Apart from inhibition of p-Jak2 and p-STAT3 in DHA-treated tumors (Fig 3C), expression of downstream proteins of STAT3 including Mcl-1, Bcl-xl, Cyclin-D1, VEGF, MMP-2, and MMP-9 was significantly suppressed in DHA-treated tumors (Fig 5E).

### DHA synergistically potentiates the antitumor activity of cisplatin in HNSCC cells

**Fig 6:**
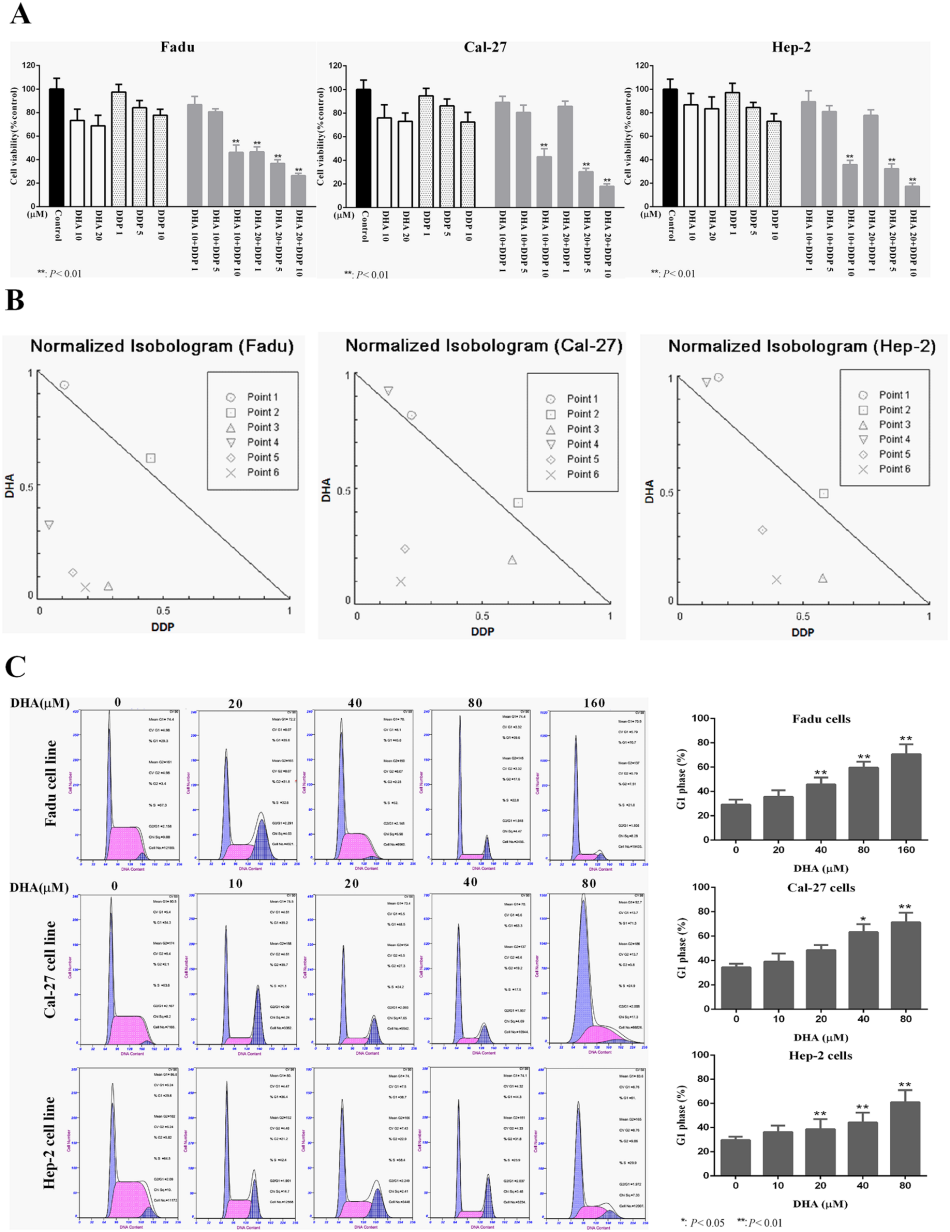
DHA synergistically potentiated cisplatin-induced proliferation inhibition and produced G0/G1 phase cell cycle arrest in HNSCC cells. (A) Fadu, Cal-27 and Hep-2 cells were treated with either DHA (10 and 20 μM), cisplatin (1, 5, and 10 μM), or a combination of both for 24 h. Proliferation inhibition was determined by MTT assay. Data expressed as means ± SD, **p<0.01. (B) Antiproliferative effects of drug synergy were determined by the CalcuSyn software (version 1.0). Combinations: point 1, DHA 10 μM and cisplatin (DDP) 1 μM; point 2, DHA 10 μM and cisplatin 5 μM; point 3, DHA 10 μM and cisplatin 10 μM; point 4, DHA 20 μM and cisplatin 1 μM; point 5, DHA 20 μM and cisplatin 5 μM; point 6, DHA 20 μM and cisplatin 10 μM. All experiments were performed in triplicates. (C) Cell cycle distribution patterns of FaDu, Cal-27 and Hep-2 cells were determined by flow cytometry after exposure to various concentrations of DHA for 24h. The proportions of cells in G1 were calculated. Data were expressed as means ± SD, *p<0.05, **p<0.01. All experiments were performed in triplicates.

The synergistic effects between the two drugs were seen in different combinations in separate cell lines (Fig 6A). For Cal-27 cells and Hep-2 cells, synergistic effects were observed when cells were treated with 10 μM DHA and 10 μM cisplatin, as well as 20 μM DHA with 5 and 10 μM cisplatin. In Fadu cells, synergistic effects were noted when cells were cultured with 10 or 20 μM DHA and5 or 10μM cisplatin. The CIs of these combinations were all less than 1, suggestive of a synergistic activity between the two drugs in proliferation inhibition on HNSCC cells (Fig 6B). Inhibition of STAT3 activation resulted in reversion of the cell cycle distribution patterns induced by upregulation of p-STAT3. It was revealed that, after treatment with DHA, a G0/G1 phase accumulation occurred in the three cell lines with a simultaneous decrease in the percentage of cells in the S and/or G2/M phase. In addition, DHA resulted in G1 cell cycle arrest in HNSCC cells in time-and dose-dependent manners (Fig 6C).

## Discussion

Searching novel therapeutic agents that target some specific signaling molecules is crucial for developing revolutionized and promising treatment modalities to cure HNSCC [21]. Of particular interest, STAT3 may serve as a potential target for this purpose. It has been demonstrated that activation of STAT3 is necessary for the growth of HNSCC cell lines [22], and the status of STAT3 activation is a marker to predict the survival and prognostic outcomes in HNSCC patients [23]. In this regard, targeting activation of STAT3 would be an ideal strategy for preventing pathogenesis and progression of HNSCC.

Up till now, several compounds have been screened and shown to inhibit the STAT3 activation. These STAT3-targeting agents are currently divided into two main groups, direct action group and indirect action group. The former group, which includes Stattic [24], ST3-H2A2 [25], S3I-1757 [26] and Diindolylmethane [27], interacts with structural domain of STAT3 and disrupts the process of STAT3 phosphorylation. By contrast, the indirect action group alters the status of STAT3 activation via acting on the upstream proteins that dominate the STAT3 activation. In fact, Pentoxifylline [28], AZD1480 [29], AG490 [30], JSI-124 (cucurbitacin I) [31], Indirubin [32] and LBH589 [33] belong to the indirect inhibitors of STAT3. Although the before-mentioned agents have been shown to possess substantial activities on inhibiting STAT3 activation, they are mostly used for experimental purposes, and only a very few have been approved for clinical trials by FDA as a STAT3 inhibitor due to major toxicity-related safety concerns. Very recently, a first-in-man phase I study was conducted in patients with refractory solid malignancies [34] to evaluate the safety, pharmacokinetics, pharmacodynamics and tumor inhibition efficacy of OPB-51602, a novel small-molecule and direct inhibitor of STAT 3 phosphorylation at Tyr705 and Ser727 sites. Although the preliminary tumor inhibition effects were observed in non-small cell lung carcinoma (NSCLC), the side effects and dose-related toxicities of the chemical were obvious, which need further in-depth and long-term clinical investigations.

As a highly effective treatment drug of falciparum malaria, DHA has been proved to be a safe, well tolerated, and widely used in clinic. Moreover, a few studies have reported that the efficient antimalarial drug DHA shares strong antitumor activities in different human cancer cells, such as colorectal carcinoma cells, T-lymphoma cells, and leukemia cells [35–37]. However, there has been no available evidence for proving the effects of DHA on growth of HNSCC cell inhibition to date. In the present study, it is for the first time we demonstrated that DHA possesses antitumor properties against a variety of human HNSCC cells both in vitro and in vivo. The underlying mechanism involves selective inhibition of Jak2/STAT3 signaling and its downstream target proteins, thereby producing proliferation inhibition and inducing cell apoptosis.

**Fig 7:**
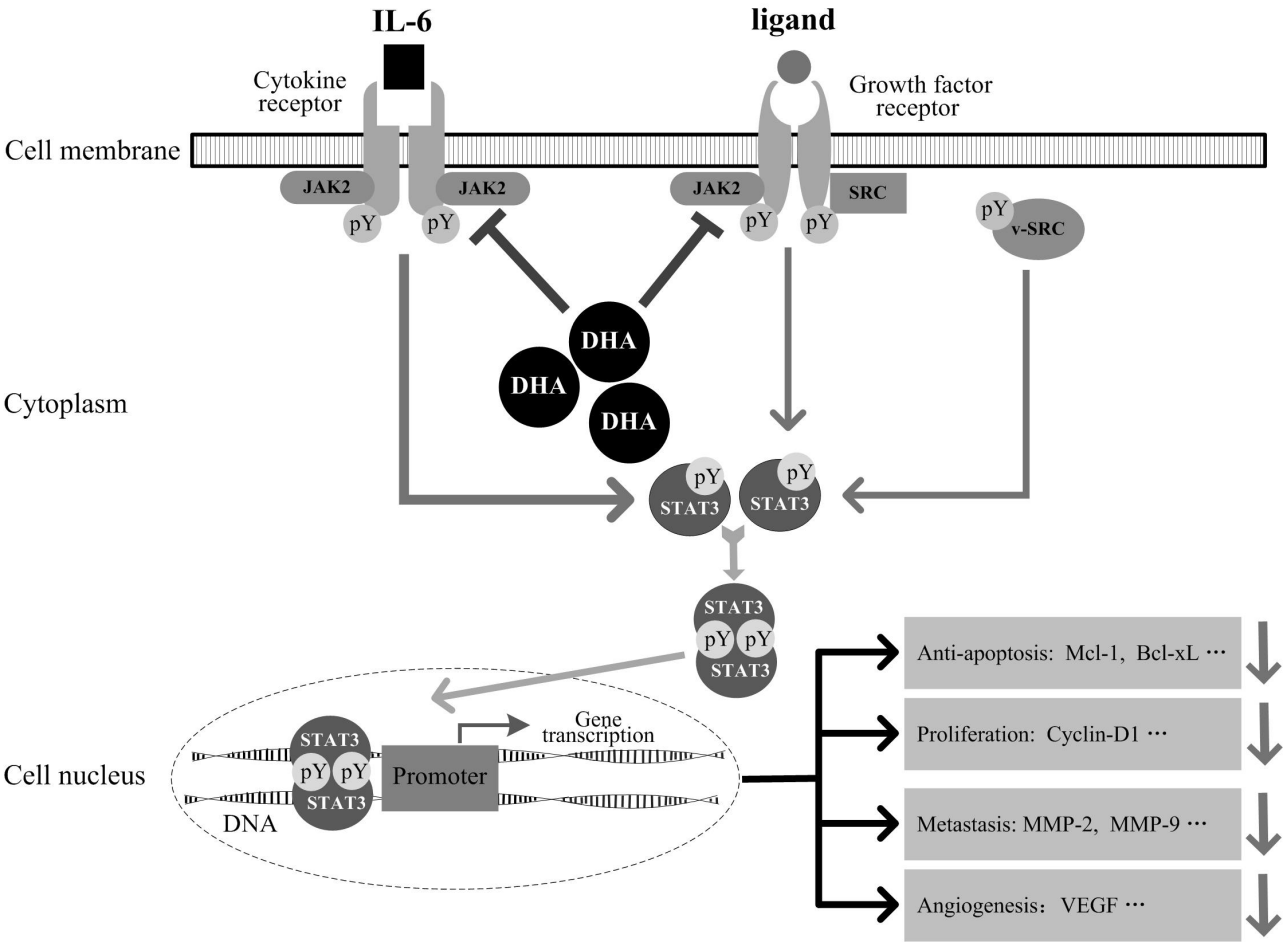
A schematic chart showing the pathways by which DHA inhibits STAT3 activation and signaling.

Our results also demonstrated that the DHA-induced inhibitory effects on STAT3 signaling are selective and specific, as we summarized in Fig 7. In the present study, we made it clear that inhibition of STAT3 by DHA depended on blockade of Jak2 kinase rather than on inhibition of EGFR tyrosine and SRC family kinases in HNSCC cells. Moreover, DHA inhibited phosphorylation of STAT3, but did not affect the constitutive activation of Akt and ERK, the key proteins of PI3K/Akt and MAPK/RAS oncogenic signaling pathways. Our observations further confirmed the notion that Jaks are central mediators of STAT3 signaling in solid tumor cells [29].

In the present investigation, the efficacy of STAT3 inhibition by DHA was also confirmed. DHA exhibited substantial effects on inhibiting STAT3 activation both in vitro and in vivo. Inhibition of STAT3 activation by DHA exerted functional impacts on HNSCC cells, including decrease in cell viability and migratory capability, G0/G1 phase cell cycle arrest and apoptosis in HNSCC cells. As postulated in Fig 7, these effects are most likely to be attributed to downregulation of the cycle regulator cyclin D1, antiapoptotic proteins Bcl-xL and Mcl-1, growth factor VEGF, and metastasis-associated proteins MMP-2 and MMP-9, most of which are major downstream targets of STAT3 [33, 38].

Apart from direct inhibition of tumor growth, blocking STAT3 activation is of potential value in combating chemoradiotherapeutic resistance of HNSCC. For instance, activation of STAT3 is associated with resistance of laryngeal carcinoma cells to ionized radiation; blockade of STAT3 signaling by shRNA sensitized the laryngeal carcinoma cells to radiotherapy both in vitro and in vivo [39]. Moreover, activation of STAT3 is also associated with chemoresistance of HNSCC [40]. In the present study, we demonstrated for the first time that DHA potentiates the antiproliferative effects of cisplatin in HNSCC cells. It would be of great interest to further elucidate whether DHA has synergized effects with other chemotherapy drugs in killing HNSCC cells.

STAT3 activation is also responsible for HNSCC resistance to some molecular targeted therapies. In fact, activation of STAT3 is related to resistance of HNSCC to EGFR monoclonal antibodies, such as Cetuximab [41]. Targeting STAT3 with a STAT3 decoy reduced cellular viability and the expression of STAT3 target genes in EGFR inhibitor (Cetuximab) resistance models; the addition of a STAT3 inhibitor to EGFR blocking strategies significantly enhanced antitumor effects of Cetuximab in vivo [42]. Activation of STAT3 also constitutes a cause of insensitivity of HNSCC cells to proteasome inhibitor. For instance, Bortezomib up-regulates STAT3 and synergizes with inhibitors of STAT3 to promote cell death in HNSCC [43]. Therefore, addition of DHA, as a putative and effective STAT3 inhibitor, to the above-mentioned targeting therapies may greatly enhance the treatment efficacies and thus outcomes of HNSCC patients.

Owing to the scarcity of the available STAT3 inhibitors that can be used in clinic, defining DHA as a putative STAT3 inhibitor is of profound clinical implications. Because our results are preclinical, further controlled clinical trials are due to carry out to confirm the efficacy of DHA in improving the outcomes of HNSCC or some other human malignancies.

## Supporting Information

S1 Fig Basal expression levels of p-STAT3 (Tyr705) and STAT3 in Fadu, Cal-27 and Hep-2 cells. (TIF)

S2 Fig The dose-escalation studies of AZD1480 and AG490 in HNSCC cells. (TIF)

S1 File RT-PCR and Western blotting of associated genes in Cal-27 cells transfected with different plasmids. 3×105 Cal-27 cells per well were seeded in 6-well plates. When plated cells reached 80% confluence, they were transiently transfected with plasmids of DN-Jak2 (Empty vector, 19017–1, 19018–1, and 19019–1), DN-EGFR (Empty vector, 7991–1, 7992–1, and 7993–1), and DN-SRC (Empty vector, 18909–1, 18910–1, and 18911–1). The transfection was performed with Lipofectamine 2000 according to the manufacturer’s instructions. After 24 h, the transfection efficiency was analyzed by RT-PCR (Figure A) and Western blotting (Figure B). The results indicated that 19019–1 (DN-Jak2), 7992–1 (DN-EGFR), and 18911–1 (DN-SRC) were the optimum plasmids to inhibit the corresponding genes. Therefore, we chose these plasmids to fulfill the transfection study in Cal-27 cells.
(TIF)
