# Supplementary figures and images for: Dihydroartemisinin as a Putative STAT3 Inhibitor, Suppresses the Growth of Head and Neck Squamous Cell Carcinoma by Targeting Jak2/STAT3 Signaling
Source: PLoS One. 2016 Jan 19;11(1):e0147157. doi: 10.1371/journal.pone.0147157 (PMC4718674; doi:10.1371/journal.pone.0147157)

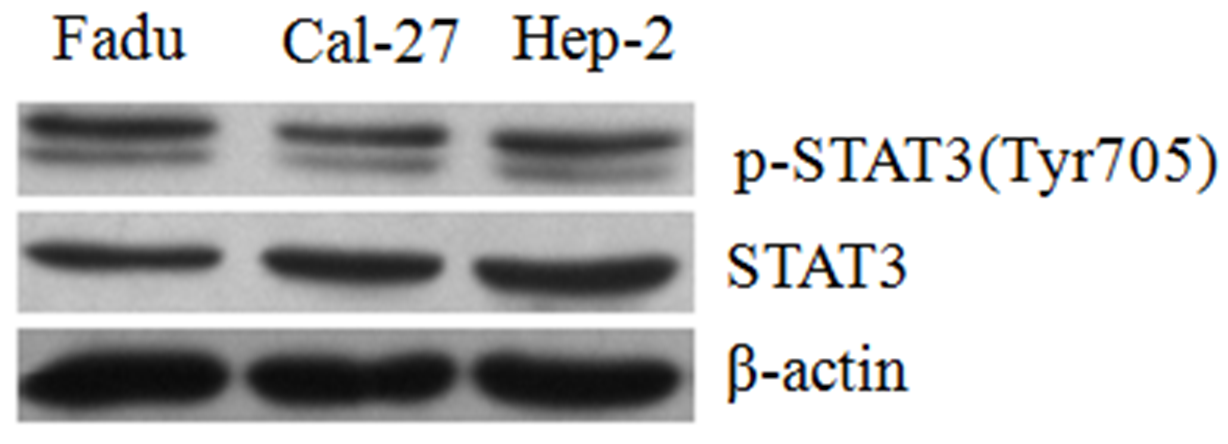

Supplement: S1 Fig — (TIF) [file pone.0147157.s001.tif]

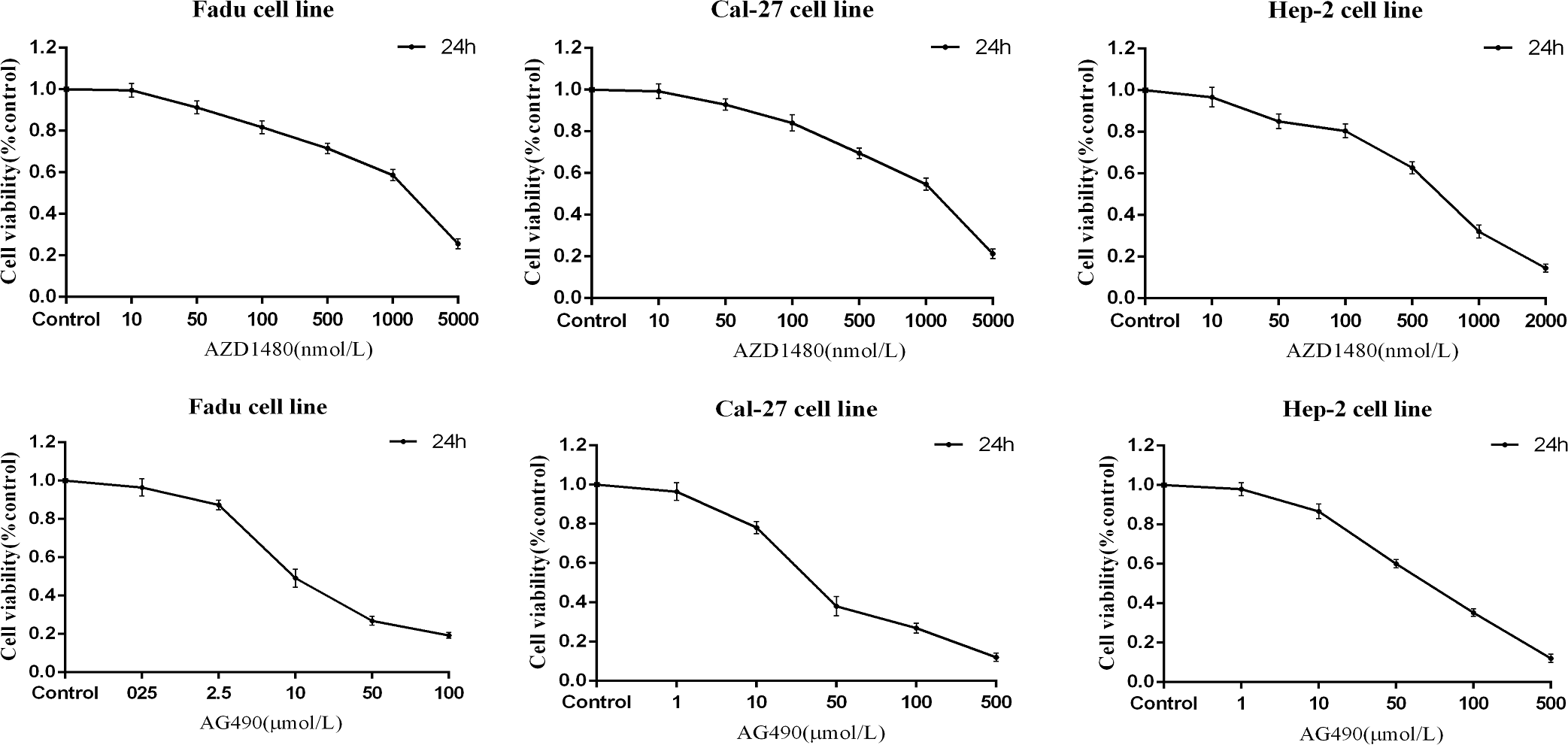

Supplement: S2 Fig — (TIF) [file pone.0147157.s002.tif]

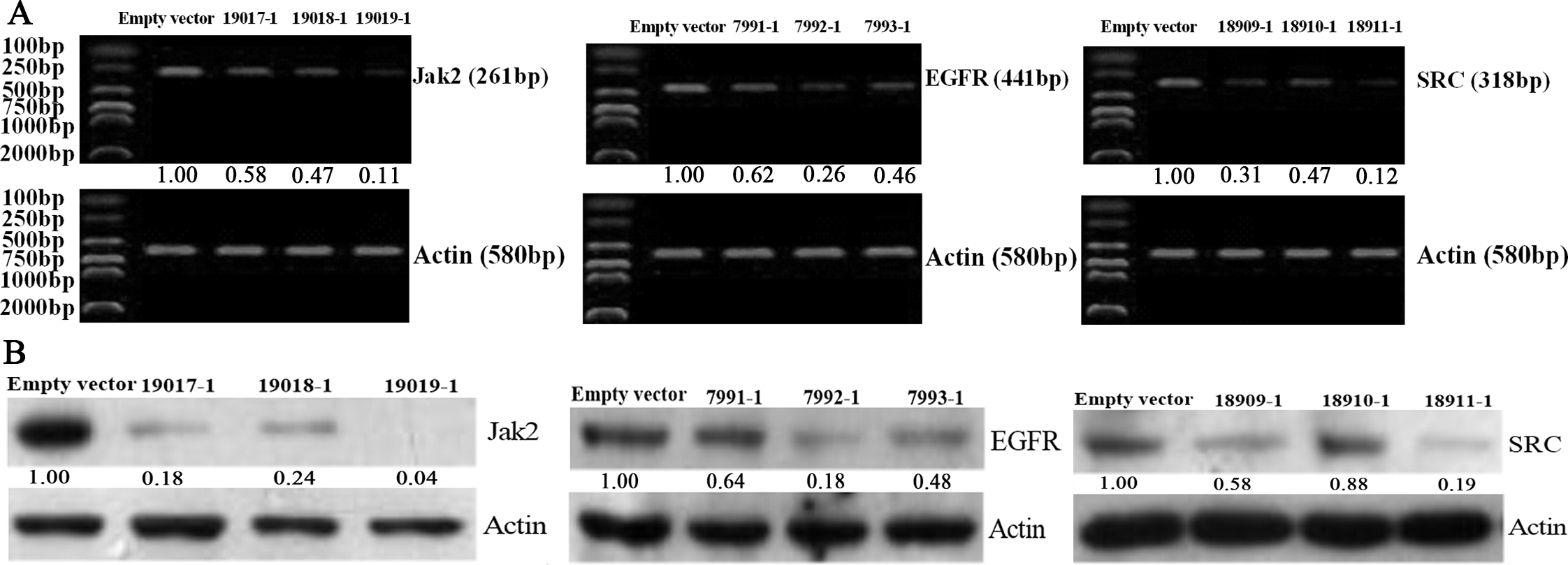

Supplement: S1 File — 3×10^5^ Cal-27 cells per well were seeded in 6-well plates. When plated cells reached 80% confluence, they were transiently transfected with plasmids of DN-Jak2 (Empty vector, 19017–1, 19018–1, and 19019–1), DN-EGFR (Empty vector, 7991–1, 7992–1, and 7993–1), and DN-SRC (Empty vector, 18909–1, 18910–1, and 18911–1). The transfection was performed with Lipofectamine 2000 according to the manufacturer’s instructions. After 24 h, the transfection efficiency was analyzed by RT-PCR (Figure A) and Western blotting (Figure B). The results indicated that 19019–1 (DN-Jak2), 7992–1 (DN-EGFR), and 18911–1 (DN-SRC) were the optimum plasmids to inhibit the corresponding genes. Therefore, we chose these plasmids to fulfill the transfection study in Cal-27 cells. (TIF) [file pone.0147157.s003.tif]
